# Decreased expression of insulin-degrading enzyme increases gluconeogenesis and glucose production in cultured hepatocytes administered with glucagon

**DOI:** 10.1038/s41598-025-03790-2

**Published:** 2025-05-31

**Authors:** Carlos M. González-Casimiro, Patricia Cámara-Torres, Beatriz Merino, Alma M. Astudillo, Miguel A. de la Fuente, Cristina M. Ramírez, Andrés Alonso, Irene Cózar-Castellano, Germán Perdomo

**Affiliations:** 1 Instituto de Biomedicina y Genética Molecular (Consejo Superior de Investigaciones Científicas-Universidad de Valladolid), c/ Sanz y Forés, 3, 47003 Valladolid, Spain; 2 Departamento de Biología Celular, Genética, Histología y Farmacología, Facultad de Medicina, Universidad de Valladolid, 47002 Valladolid, Spain; 3 Centro de Investigación Biomédica en Red de Diabetes y Enfermedades Metabólicas Asociadas (CIBERDEM), 28029 Madrid, Spain; 4 IMDEA Research Institute of Food & Health Sciences, Madrid, Spain

**Keywords:** Glucagon, Gluconeogenesis, Hepatocytes, Insulin-degrading enzyme, Hormones, Cell signalling

## Abstract

Insulin-degrading enzyme (IDE) is a protein with proteolytic and non-proteolytic functions that regulates glucose homeostasis. In the fasted state, glucagon regulates glycemia through induction of hepatic gluconeogenesis. The rate of hepatic gluconeogenesis is elevated in subjects with type 2 diabetes (T2D) compared with healthy subjects. Interestingly, subjects with T2D show decreased expression of hepatic IDE. However, the role of IDE on the regulation of hepatic gluconeogenesis is completely unknow. We hypothesize that IDE deficiency alters glucagon signaling and thereby gluconeogenesis. To test this hypothesis, we used mouse liver tissues and cultured hepatocytes with total or partial IDE deficiency. The glucagon signaling pathway, expression of gluconeogenic genes, glucose production, and transcriptomic analysis were performed in control and IDE-KO hepatocytes. Total or partial loss of IDE in liver tissues or cultured mouse hepatocytes resulted in lower levels of the glucagon receptor (GCGR) and the cAMP-response element binding protein (CREB). However, glucagon stimulation increased the phosphorylation of CREB, despite lower levels of cAMP in IDE-deficient mouse hepatocytes. The activation of CREB was associated with an upregulation of the gluconeogenic genes *Pck1* and *G6pc* (~ 200% and ~ 70% respectively) and higher glucose production in IDE-deficient mouse hepatocytes. Finally, genetic depletion of IDE in HepG2 hepatocytes led to upregulation of genes involved in cellular functions related to membranes, organelles and signaling receptors. These findings may be of relevance to better understand the regulation of hepatic gluconeogenesis and the use of IDE as a potential therapeutic target for the treatment of T2D.

## Introduction

Insulin-degrading enzyme (IDE) is a neutral Zn^2+^ metallo-endopeptidase ubiquitously expressed that can degrade different substrates, including insulin and glucagon^1,2^. Genetic polymorphisms within the *Ide* locus are associated with increased risk of type 2 diabetes (T2D)^3^. Furthermore, serum IDE levels are elevated in subjects with T2D or metabolic syndrome compared to metabolically healthy individuals^4,5^, which has suggested that IDE may be a biomarker to identify individuals at risk to develop T2D.

Gluconeogenesis is an important cellular process by which the body synthesizes glucose from non-carbohydrate sources. This is a vital physiological process for maintaining blood glucose levels, particularly during fasting or when carbohydrate intake is insufficient^6,7^. In addition, in pathological conditions, such as T2D, abnormally increased hepatic gluconeogenesis is a significant contributor to hyperglycemia in the fasting state^8^.

In recent decades, scientists have made significant progress in deciphering molecular mechanisms by which glucagon regulates gluconeogenesis in response to the fasted state. Thus, glucagon binds to its receptor (GCGR) on the plasma membrane of the cell, allowing a conformational change that activate G protein coupled-receptor (GPCR). As a consequence, adenylate cyclase is activated leading to increases in cAMP levels, which in turn stimulates activation of protein kinase A (PKA) leading to the phosphorylation of the cAMP response element-binding (CREB) protein. The transcriptional factor CREB is responsible for inducing the transcription of the gluconeogenic genes glucose-6-phosphatase (*G6pc*) and phosphoenolpyruvate carboxykinase (*Pck1*). In this way, glucagon facilitates the cellular response to fasting, leading to hepatic gluconeogenesis and glucose release^9–11^. For several decades this “canonical pathway” mediated by the Gαs subunit of the heterotrimeric G protein complex has been the prevailing view. More recently, evidence supports and alternative glucagon signaling pathway involving the Gαq protein-coupled receptor subunit that activates phospholipase C (PLC) and catalyzes the formation of inositol-1,4,5-triphosphate (IP3) and diacylglycerol (DAG) from phosphatidylinositol-4,5-bisphosphate (PIP2). IP3 production promotes intracellular calcium efflux into cytosol leading to transcriptional activation of gluconeogenic genes independent of cAMP levels^9,12^.

The role of IDE as a potential regulator of the intracellular glucagon signaling pathway and its downstream targets is completely unknown. Interestingly, in T2D subjects it has been observed a decreased hepatic *Ide* expression^13^. In mice, high-fat feeding resulted in ~ 30% reduction of hepatic IDE levels during fasting^14^. Finally, it has been proposed that glucagon increases IDE levels via cAMP/PKA-dependent pathway in Hepa-1c1c7 cells^15^. These observations led us to hypothesize that IDE may participate in the glucagon-mediated regulation of gluconeogenesis in hepatocytes. In this work, we aimed to decipher the role of IDE in the regulation of glucagon signaling and gluconeogenesis in hepatocytes.

## Results

### Genetic depletion of IDE in the liver of mice (L-IDE-KO) altered glucagon signaling and upregulated gluconeogenesis

**Fig. 1:**
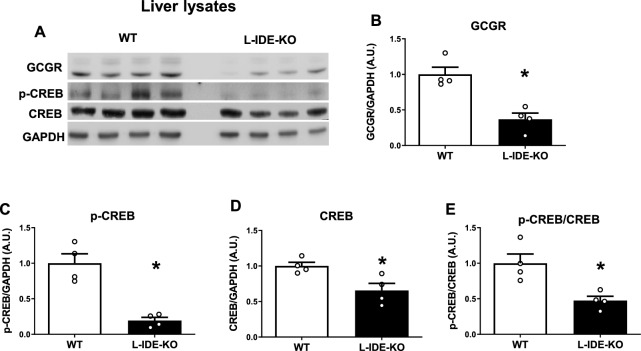
Effects of liver-specific depletion of IDE on the glucagon signaling pathway. Livers from fasted 3-month-old WT and L-IDE-KO mice were excised and protein lysates were prepared by homogenizing the tissues in lyses buffer. (**A**) Representative western blot of liver lysates (40 µg protein/sample) from WT (white bars) or L-IDEKO (black bars). (**B**) Densitometric analyses of the data in panel A of the glucagon receptor (GCGR), (**C**) p-CREB, (**D**) CREB and (**E**) the ratio p-CREB versus total CREB protein. Data are expressed relative to WT. Mean ± SEM for n = 4 independent experiments per genotype. *p value < 0.05 versus WT by Students´ T-test.

We have previously described that fasted L-IDE-KO mice have increased expression of gluconeogenic genes (*Pck1* and *G6pc*) than control mice despite similar circulating glucagon levels^16^, suggesting that hepatic IDE may play a role on the regulation of glucagon signaling. To elucidate the molecular basis underlying this phenotype, we analyzed multiple components of the intracellular signaling pathway in lysates obtained from frozen livers of fasted WT and L-IDE-KO mice^16^. As seen in Fig. 1, loss of hepatic IDE resulted in a significant decreased (~ 50–60%) in the glucagon receptor (GCGR) and the cAMP-response element binding protein (~ 30–40%) (CREB) levels. Furthermore, phosphorylation of CREB (p-CREB) was reduced by ~ 80% in L-IDE-KO mice, compared to controls (Fig. 1C-E). These data suggest that liver-specific ablation of IDE may regulate glucagon signaling in mouse hepatocytes.

### Genetic depletion of IDE altered glucagon signaling and upregulated gluconeogenesis in response to glucagon in mouse primary hepatocytes

**Fig. 2:**
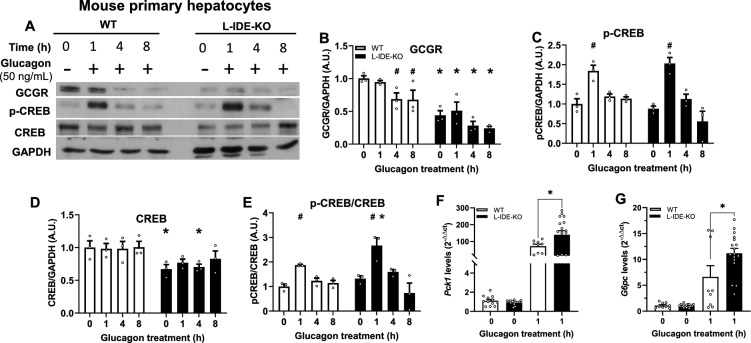
Effects of genetic depletion of IDE on glucagon signaling and expression of gluconeogenic genes in primary mouse hepatocytes. Primary hepatocytes isolated from fasted 3-month-old WT and L-IDE-KO mice were treated with glucagon (50 ng/mL) at the indicated times followed by quantification of protein levels of the glucagon signaling pathway. (**A**) Representative western blot (40 µg protein/sample) depicting WT (white bars) and L-IDE-KO (black bars) mouse primary hepatocytes treated with glucagon. Densitometric analyses of the data in panel A of the GCGR (**B**), p-CREB (**C**), CREB (**D**) and the ratio p-CREB versus total CREB protein (**E**). Data are expressed relative to WT. Mean ± SEM for n = 3 independent experiments per genotype. ^*^p value < 0.05 versus WT by two-way ANOVA. ^#^*p* value < 0.05 versus untreated cells (time 0) by two-way ANOVA. Primary hepatocytes isolated from WT and L-IDE-KO mice were treated with glucagon as above followed extraction and quantification of mRNA levels of *Pck1* (**F**) and *G6pc* (**G**). Data are mean ± SEM (relative to control) for n = 3–6 independent experiments in triplicate per genotype and condition. *p value < 0.05 versus WT by Students’ T-test.

To investigate a potential role of IDE in the regulation of hepatic glucagon signaling, we isolated mouse primary hepatocytes from WT and L-IDE-KO mice. Hepatocytes were serum starved overnight prior to glucagon stimulation (50 ng/mL) in a time-dependent manner as indicated in Fig. 2. The intracellular glucagon signaling pathway and expression of gluconeogenic genes were studied. As expected, glucagon treatment (1 h) increased the phosphorylation of CREB (~ twofold) in WT hepatocytes by without changes in the protein levels of GCGR and CREB (Fig. 2A–E). This stimulation of the signaling pathway resulted in the upregulation of the gluconeogenic genes *Pck1* and *G6pc* (Fig. 2F,G). Of note, prolonged (4–8 h) stimulation with glucagon reduced by ~ 30% GCGR levels in control hepatocytes, suggesting a time-dependency activation of the signaling pathway (Fig. 2A,B).

However, hepatocytes isolated from L-IDE-KO mice showed a significant reduction in the GCGR (~ 50%) and CREB (~ 30%) levels before glucagon treatment as compared to WT hepatocytes (Fig. 2B,D). These findings resemble those observed in the liver of fasted L-IDE-KO mice (Fig. 1B,D).

A puzzling finding was the observation that the ratio p-CREB/CREB in hepatocytes from L-IDE-KO mice was higher (~ 33%) than in hepatocytes from WT mice after glucagon treatment (1 h) (Fig. 2E), despite lower levels of the GCGR and CREB. This activation of CREB paralleled an increased expression of gluconeogenic genes *Pck1* and *G6pc* (~ 200% and ~ 70% respectively) in L-IDE-KO hepatocytes compared to WT hepatocytes (Fig. 2F,G).

### IDE deficiency altered glucagon signaling and upregulated gluconeogenesis in response to glucagon in mouse AML12 hepatocytes

**Fig. 3:**
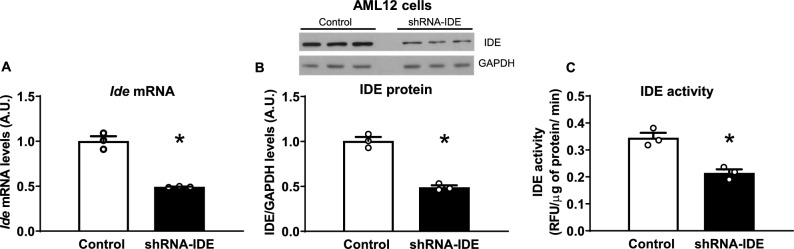
Development and characterization of a mouse IDE knockdown cell line. Mouse hepatocytes (AML12) were transfected with an shRNA to knockdown *Ide*. (**A**) mRNA levels, (**B**) representative western blot image and quantification of the protein levels, and (**C**) assessment of the proteolytic activity of IDE. Results are mean ± SEM. n = 3 per group. *p value < 0.05 versus control by Students’ T-test.

To further investigate the impact of IDE deficiency on glucagon signaling and gluconeogenesis in hepatocytes, we knocked-down *Ide* in AML12 cells using a lentivirus containing a shRNA-IDE. This approach led to ~ 50% reduction in IDE mRNA and protein levels (Fig. 3A,B) and in its proteolytic activity (Fig. 3C).

**Fig. 4:**
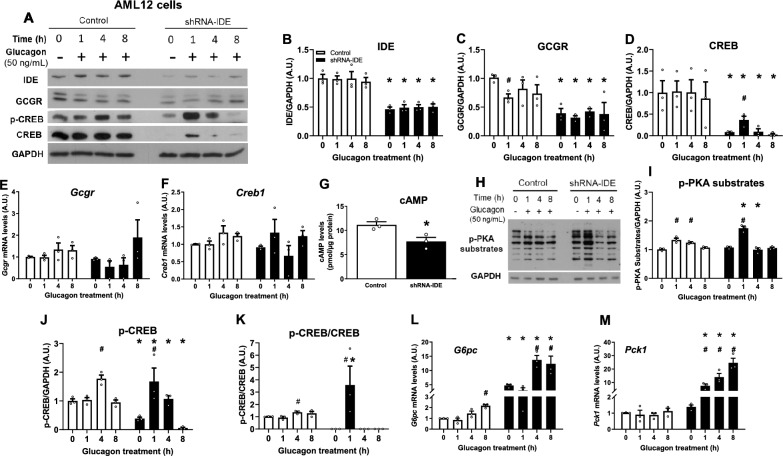
Effects of IDE deficiency on glucagon signaling and expression of gluconeogenic genes in a cell line of hepatocytes. AML12 cells were serum-starved for 18 h followed by incubation with glucagon (50 ng/mL) at the indicated times and the effects of *Ide* deficiency on glucagon signaling and expression of gluconeogenic genes were examined. (**A**) Representative western blots depicting control (white bars) and shRNA-IDE (black bars) hepatocytes treated with glucagon. Densitometric analysis of data in panel A for IDE (**B**), GCGR (**C**) and CREB (**D**). Data are mean ± SEM. n = 3 per group. ^#^*p* value < 0.05 versus untreated cells (time 0) or *p value < 0.05 versus control cells by two-way ANOVA. Gene expression levels of *Gcgr* (**E**) or *Creb1* (**F**). Data are mean ± SEM. n = 3 per group. ^#^*p* value < 0.05 versus untreated cells (time 0) or *p value < 0.05 versus control cells by two-way ANOVA. (**G**) cAMP levels after 30 min of glucagon stimulation in control and IDE-deficient cells. Data are mean ± SEM. n = 3 per group. *p value < 0.05 versus control cells by Students´ T-test. (**H**) Representative western blots of p-PKA substrates for control and shRNA-IDE cells treated with glucagon (50 ng/mL) at the indicated times. (**I**) Densitometric analysis of data in panel H for p-PKA substrates. Data are mean ± SEM. n = 3 per group. ^#^*p* value < 0.05 versus untreated cells (time 0) or *p value < 0.05 versus control cells by two-way ANOVA. (**J**) Densitometric analysis of data in panel A for p-CREB and the ratio p-CREB/CREB (K). Data are mean ± SEM. n = 3 per group. ^#^*p* value < 0.05 versus untreated cells (time 0) or *p value < 0.05 versus control cells by two-way ANOVA. Gene expression levels of *G6pc* (L) or *Pck1* (M). Data are mean ± SEM. n = 3 per group. ^#^*p* value < 0.05 versus untreated cells (time 0) or *p value < 0.05 versus control cells by two-way ANOVA.

In control cells, glucagon augmented the production of cAMP and activation of p-PKA substrates leading to increased phosphorylation of CREB and upregulation of the gluconeogenic gene *G6pc* (Fig. 4). In this study model, cAMP production and stimulation of p-PKA substrates was reached at 1 h of glucagon stimulation, but p-CREB and expression of the gluconeogenic genes was observed between 4 and 8 h.

However, IDE-deficient hepatocytes (~ 50%) showed a significant reduction in the GCGR (~ 60%) and CREB (~ 90%) levels before glucagon preincubation as compared to control cells (Fig. 4B,D). Most likely, the lower levels of the GCGR and CREB were not related to transcriptional regulation (Fig. 4E,F). Deficiency of IDE in cultured hepatocytes was associated with lower GCGR and CREB levels, similarly to what was observed in the liver tissues and hepatocytes isolated from the L-IDE-KO mice (Figs. 1B,D and 2B,D respectively).

As expected from lower GCGR levels, the generation of cAMP was diminished (~ 30%) in shRNA-IDE hepatocytes compared to control cells (Fig. 4G). Surprisingly, despite a significant reduction in the GCGR and cAMP levels, glucagon stimulation (1 h) increased the number of substrates phosphorylated by PKA (p-PKA substrates) in IDE-deficient cells compared to control cells (Fig. 4H). Coinciding with higher levels of p-PKA substrates, the ratio p-CREB/CREB (1 h) was significantly higher in IDE-deficient hepatocytes compared to control cells (Fig. 4K). The glucagon-mediated phosphorylation of CREB resulted in ~ 15 to ~ 25-fold increase (1–4 h) in the expression of the gluconeogenic genes *G6pc* and *Pck1* as compared to control cells (Fig. 4L,M). Taken together, these data demonstrate that glucagon upregulates the expression of gluconeogenic genes in IDE-deficient hepatocytes.

Finally, GCGR can also signal through the Gαq protein-coupled receptor subunit that activates phospholipase C (PLC) and catalyzes the formation of inositol-1,4,5-triphosphate (IP3) and diacylglycerol (DAG) from phosphatidylinositol-4,5-bisphosphate (PIP2). Glucagon stimulation of IDE-deficient hepatocytes showed similar amounts of total DAG as compared to control cells, suggesting that the knock down of IDE does not alter this arm of the glucagon signaling pathway (Supplementary Fig. 1A–E).

### The cytoplasmic isoform of IDE may regulate glucose production in mouse AML12 hepatocytes

There are two isoforms of IDE (mitochondrial and cytoplasmic), which are formed by alternative translation initiation encoding proteins beginning either at the first (Met^1^-IDE) or the 42^nd^ amino acid (Met^42^-IDE). The cytoplasmic isoform (Met^42^-IDE) is predominantly expressed in tissues and cultured cells, because of the existence of a better Kozak consensus sequence for initiation of translation^17^. The specific function of the cytoplasmic isoform in the regulation of intracellular signal transduction is unknown. We hypothesized that the cytoplasmic isoform would be responsible for the regulation of glucose production in AML12 cells.

**Fig. 5:**
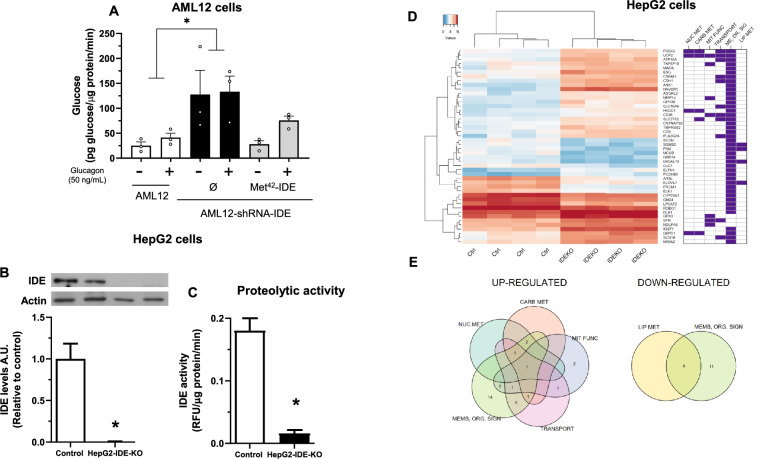
Effect of the cytoplasmic IDE on glucose production and transcriptomic analysis in IDE-deficient hepatocytes. The cytoplasmic isoform of IDE regulates glucose production in hepatocytes. (**A**) AML12-shRNA-IDE cells were transfected with a plasmid containing the cytoplasmic isoform of IDE (Met^42^-IDE) and stimulated with glucagon (50 ng/mL) for 1 h. Afterwards, glucose production was quantified. Results are mean ± SEM. n = 3 per condition. *p value < 0.05 versus control by two-way ANOVA. Characterization of HepG2 cells with genetic depletion of IDE. HepG2-IDE-KO cells were generated using the CRISPR/Cas9 as described in the Methods section. (**B**) IDE protein levels. Upper panel, representative western blot image. Lower panel, quantification of IDE in control and HepG2-IDE-KO cells. Results are mean ± SEM. n = 4 per group. *p value < 0.05 versus control by Students’ T-test. (**C**) Assessment of the proteolytic activity of IDE in control and HepG2-IDE-KO cells. Results are mean ± SEM. n = 3 (control) and n = 5 (HepG2-IDE-KO). *p value < 0.05 versus control by Students´ T-test. Transcriptomic analyses in HepG2 cells. (**D**) Colored heatmap (left) shows gene expression (blue: low expression, red: high expression) across control and HepG2-IDE-KO samples. Right panel shows a functional profile of differentially expressed genes (NUC MET: nucleotides metabolism; CARB MET: carbohydrates metabolism; MIT FUNC: mitochondrial function; ME, OR, SIG: Membrane, organelles, signaling receptor; LIP MET: lipids metabolism). (**E**) Venn diagram. Number of up-regulated (left) and down-regulated (right) genes sharing one or more functional profiles. Genes with an adjusted *p*-value < 0.05 and a log2(Fold Change) > 1 were considered significantly differently expressed.

To test this hypothesis, AML12-shRNA-IDE cells were transfected with a plasmid containing the cytoplasmic isoform of IDE (Met^42^-IDE) followed by assessment of glucose production. As expected, IDE-deficiency resulted in augmented glucose production (Fig. 5A). However, reconstitution of AML12-shRNA-IDE cells with the cytoplasmic IDE isoform lowered glucose production to similar levels than control cells, although this effect did not achieve statistical significance (Fig. 5A). These results suggest that the cytoplasmic isoform of IDE may be of relevance for the control of glucose production in cultured hepatocytes.

### 2.5 Transcriptome analysis of differentially expressed genes induced by genetic depletion of IDE in HepG2 cells provides insight into the role of IDE in different cellular functions

To better understand how genetic depletion of IDE plays some role in controlling different cellular functions in cultured hepatocytes, we performed transcriptomic analyses in human hepatocytes lacking IDE (HepG2-IDE-KO). Genetic ablation of *Ide* resulted in complete loss of IDE protein and activity in HepG2 cells (Fig. 5B,C). As illustrated in Fig. 5D,E, loss of IDE mainly upregulates a wide set of gene´s transcripts involved in cellular functions related to membranes, organelles and signaling receptors compared to control cells, suggesting a profound remodeling of receptors trafficking and signaling pathways.

Transcriptomic analyses also uncovered novel aspects of how IDE regulates hepatic gluconeogenesis. Thus, hexokinase domain containing 1 (HKDC1), a hexokinase that converts glucose to glucose-6-phosphate (G6P) was upregulated ~ 2.2-fold. In addition, glucose-6-phosphatase catalytic subunit 1 (G6PC1), that catalyzes hydrolysis of G6P to glucose, at the terminal step of the gluconeogenesis, was upregulated ~ 3.3-fold.

Interestingly, gene expression of the G protein subunit gamma 4 (GNG4) was downregulated by ~ twofold, suggesting an impaired regulation of the GPCR heterotrimer, which may result in lower activation of adenylate cyclase. Furthermore, the gene expression of phosphodiesterase 4D (PDE4), an enzyme responsible for the degradation of cAMP, was elevated by ~ 2.7-fold, which may also contribute to diminish cAMP levels and signal transduction.

## Discussion

In this study, we have investigated the effects of IDE on the regulation of the glucagon signaling pathway in hepatocytes. First, we identified that genetic depletion of IDE in the liver of mice (L-IDE-KO) resulted in reduced expression of the GCGR and the transcriptional factor CREB. Second, these results were confirmed in primary mouse hepatocytes isolated from L-IDE-KO mice. Third, partial loss of IDE in mouse hepatocytes (AML12 cells) recapitulated the findings seen in liver tissues and primary mouse hepatocytes. Thus, we revealed that IDE is a novel regulator of proteins that participate in the intracellular transduction of the glucagon signal.

Historically, IDE has been considered a promiscuous protease that degrades insulin and glucagon, among other small peptides^1^, although non-proteolytic functions have been described, e.g. IDE inhibits α-synuclein fibril formation by binding to α-synuclein oligomers^18^. Special attention has been paid to its role in the hepatic insulin clearance. The scientific literature supports the notion that IDE regulates circulating levels of insulin in vivo^3^. For example, chromium-insulin (a novel form of insulin directly conjugated with chromium instead of zinc) reduces insulin clearance by inhibiting hepatic IDE protein expression in KKAy mice^19^. Loss of hepatic IDE function in aging was associated with lower hepatic insulin clearance^20^. But this proteolytic function is not without controversy because loss or gain of IDE function did not alter hepatic insulin clearance in normal and diet-induced obese mice^16,21^. Thus, our findings on IDE regulation of intracellular glucagon signalling open novel avenues to explore other IDE functions beyond its proteolytic activity.

Early reports showed that long-acting glucagon preparations decreased GCGR in liver plasma membranes of treated rats without altering affinity or kinetics of association^22^. Factors, which are known to raise intracellular cAMP levels, such as 3-isobutyl-1-methylxanthine, isoproterenol, and forskolin, all caused a reduction in glucagon receptor mRNA expression in cultured rat hepatocytes^23^. In models of high-fat diet-induced hepatic steatosis, decreased glucagon receptor expression was observed in hepatocytes, suggesting obesity-associated hepatic resistance to glucagon^24^. Finally, glucagon induces internalization and recycling of the GCGR, and prolonged exposures or high concentrations of glucagon direct the receptor toward lysosomal degradation^25^. In this work, we showed that the lower levels of GCGR observed in cultured hepatocytes were most likely not related to transcriptional regulation. A plausible explication of our results may be due to the proposed role of IDE in the regulation of the proteasome^26–28^. Thus, total or partial deficiency of IDE would enhance proteasomal degradation of the GCGR. However, Krilov et al. showed that GCGR degradation was inhibited by lysosomal, but not proteasomal inhibitors^25^. Finally, here we showed that genetic depletion of IDE in hepatocytes mainly upregulated genes involved in cellular functions related to membranes, organelles and signaling receptors, suggesting a profound remodeling of receptors trafficking and their signaling pathways. Therefore, a possible explanation for the reduction in GCGR levels may be due to defects in the cellular machinery that regulates its internalization and recycling to the plasma membrane. In support of this notion, we have previously demonstrated that loss of IDE (L-IDE-KO mouse) diminished the insulin receptor protein levels in hepatocytes^16^. Nonetheless, further research is warranted to decipher the role of IDE in the regulation of intracellular trafficking of plasma membrane receptors.

Total or partial loss of IDE also resulted in lower protein levels of CREB. Most likely, the lower levels of CREB observed in the three study models of this work were not related to transcriptional regulation. To our best of knowledge, this is the first report showing regulation of CREB protein levels. Interestingly, Erion et al. showed that decreased CREB expression (by means of antisense oligonucleotide) in four different rodent models of insulin resistance reduced hepatic lipid accumulation and improved insulin sensitivity, suggesting that CREB plays a role in the regulation of hepatic lipid metabolism^29^. Here, we show that CREB levels were diminished in the liver of L-IDE-KO mice. We reported that these mice showed lower hepatic accumulation of triglycerides and increased expression of CPTI, the key enzyme that regulates the entry of long-chain fatty acids into mitochondria for subsequent β-oxidation^21^. Thus, the lower CREB protein levels may be a mechanistic explanation for the hepatic lipid metabolism seen in the L-IDE-KO mouse model.

Another aim of this work was to investigate how total or partial loss of IDE impact on hepatic gluconeogenesis. We have previously observed that genetic depletion of hepatic IDE in fasted mice (L-IDE-KO) was associated with increased expression of gluconeogenic genes^16^. Here, we show that upon glucagon stimulation, in a time-dependent manner, primary mouse hepatocytes isolated from L-IDE-KO mice and IDE-deficient AML12 cells resulted in higher expression of gluconeogenic genes. These study models (ex vivo and in vitro) recapitulated our previous findings observed in vivo (L-IDE-KO mouse)^16^. At the mechanistic level, how total or partial loss of IDE can increase the expression of gluconeogenic genes remains to be deciphered. Kupfer et al. suggested that IDE interacts directly with and enhances the DNA binding of androgen and glucocorticoid receptors, which may have important implications for hormones- and steroid-mediated signaling^30^. More recently, Yuan et al. have shown that inhibition of IDE by 6bK suppressed osteoclastic-specific genes at both transcriptional and translational levels affecting osteoclast differentiation and function^31^. Furthermore, in HepG2 cells, siRNA-IDE knockdown changed the expression of genes involved in cell cycle and apoptosis pathways^13^. Notably, the transcriptome profile of HepG2 cells with partial depletion of IDE^13^ and with total loss of IDE (this work) differ substantially, suggesting an IDE dose-dependent effect. Therefore, evidence in the scientific literature support the notion that IDE may be a regulator of transcriptional factors and/or gene expression, but this idea awaits further demonstration.

We found that phosphorylation of PKA-dependent substrates (including the transcriptional factor CREB) was augmented in IDE deficient hepatocytes. Several mechanisms have been reported by which PKA can be activated independent of cAMP levels^32–35^. For example, the S100 calcium binding protein A1 (S100A1) interacts with type 2 regulatory subunits of PKA leading to its activation^34^. Thus, it is plausible hypothesize that in our models of study loss of IDE may result in the activation of PKA in an independent way of cAMP levels.

The development of IDE inhibitors has been of considerable interest as a pharmacological approach for the treatment of T2D^3^. Maianti and collaborators developed a physiologically active IDE inhibitor (6bk) in mice. Acute IDE inhibition improved oral glucose tolerance in both lean and diet-induced obese mice. In contrast, IDE inhibition during intraperitoneal glucose administration resulted in impaired glucose tolerance in both mouse models^36^. Furthermore, inhibition of IDE during intraperitoneal glucose administration increased substantially the levels of glucagon, providing a possible explanation for impaired glucose tolerance during intraperitoneal injection. Thus, therapeutic approaches based on IDE inhibition awaits further confirmation. Although our results have been obtained using cellular models, if translated into the clinical setting of T2D, casts considerable doubts on the idea that therapies based on IDE inhibitors are a good approach for the treatment of T2D.

Finally, we acknowledge some limitations in our study. First, the low sample number in some experiments avoid us to draw definitive conclusions. Second, we used a single dose of glucagon, although this concentration (50 ng/mL) is close to the physiological range described in the hepatic portal system of humans, rats, dogs and pigs^12^. Third, the observed phenotypes may be the result of cellular adaptations to a complete loss of IDE, albeit partial loss of IDE in AML12 cells showed similar phenotype than complete IDE loss.

In conclusion, IDE is involved in the regulation of the gluconeogenesis probably through interactions with the glucagon signaling pathway in cultured hepatocytes.

## Methods

### Mice and primary mouse hepatocytes studies

Mice were housed in ventilated cages under a 12:12-h light–dark cycle, and water ad libitum, at the animal facility of the University of Valladolid (UVa). Mice were fed regular chow diet ad libitum. L-IDE-KO mice were generated and genotyped as we have previously described^16^. The Animal Care and Use Committee of the UVa approved all experiments (protocol #8608731). All experiments were performed in accordance with EU guidelines and regulations. Authors complied with the ARRIVE guidelines.

For experiments described in Fig. 1, fasted 12- to 14-week-old male littermate control and L-IDE-KO mice^16^ were euthanized by inhalation of isoflurane at an overdose concentration, administered via the open-drop method, before livers were dissected and snap-frozen in liquid nitrogen. Tissues were kept at − 80 °C until lysates were obtained and western blot analyses performed.

For studies using primary cultured mouse hepatocytes, non-fasting 12- to 14-week-old male littermate control and L-IDE-KO mice were isoflurane-anesthetized before hepatocytes were isolated. Then, mice were perfused with warm perfusion solution [calcium-free Hank´s balanced salt solution (HBSS), 10 mM HEPES pH 7.4, 0.2 mM EGTA] followed with collagenase digestion (Sigma-Aldrich, St. Louis, MO, USA). After digestion, hepatocytes were released by mechanical dissociation of the tissue and underwent to filtration with attached medium (DMEM:F12 supplemented with 10% FBS, 100 U/mL penicillin, 100 g/mL streptomycin) and centrifugation at 50 × *g* for 5 min. Pellets containing cells were resuspended in attached medium and loaded onto isotonic Percoll solution [40% (v/v)] (Thermo-Fisher, Waltham, MA, USA) and centrifuged at 200 × *g* for 10 min. at room temperature. Supernatants were discharged and cells were washed with attached medium. Afterwards, cells were seeded at 75,000–125,000 cells/mL on 6-well collagen-coated plates (Nunclon™ Delta Surface, Thermo-Fisher, Waltham, MA, USA) and cultured in DMEM:F12 medium supplemented with 10% FBS (Thermo-Fisher, Waltham, MA, USA), 100 U/mL penicillin, 100 g/mL streptomycin digestion (Sigma-Aldrich, St. Louis, MO, USA) for 24 h. Then, primary hepatocytes were serum starved for 18 h, preincubated with glucagon (50 ng/mL) at indicated times and harvested for protein or gene expression analyses.

### Cell cultures and glucose production assay

Mouse AML12 cells (ATCC CRL-2254, VA, USA; a cell line established from hepatocytes from a mouse transgenic for human TGFα) were cultured in DMEM:F12 medium supplemented with 10% FBS, 100 U/mL penicillin, 100 g/mL streptomycin, 10 µg/mL insulin, 5.5 µg/mL transferrin, 5 ng/mL selenium, and 40 ng/mL dexamethasone (Sigma-Aldrich, St. Louis, MO, USA) at 37 °C with 5% CO2. HepG2 cells (ATCC HB-8065, VA, USA; derived from a liver hepatocellular carcinoma of a 15-year-old Caucasian male) were cultured in Minimum Essential Medium (MEM) supplemented with 10% FBS, 100 U/mL penicillin and 100 g/mL streptomycin (Sigma-Aldrich, St. Louis, MO, USA).

Glucose production in AML12 cells was assessed using the High Sensitive Glucose Assay Kit (#MAK543 Sigma-Aldrich, USA) following manufacturer’s instructions. Briefly, cells were serum starved overnight and incubated in the presence or absence of glucagon (50 ng/mL) for 1 h in a Krebs buffer (500 µL) supplemented with 20 mM lactate and 2 mM pyruvate as a carbon source. Afterwards, Krebs buffers were collected for glucose assessments.

Serial dilutions (1:3) of a glucose Standard Stock Solution (800 mM) were performed in Assay Buffer to obtain a typical glucose standard curve. Then, 50 µL of each glucose standard, test sample blank, or blank was mixed with 50 µL of Assay Working Solution (to make the total assay volume of 100 µL/well) into each well of a black flat bottom 96-well plate. Reactions were incubated for 10–30 min at 37 °C, protected from light. To determine glucose concentration in samples, the fluorescence intensity was monitored with a fluorescence plate reader at λ_Ex/Em_ = 530–570 nm/590–600 nm (optimal λ_Ex/Em_ = 540/590 nm).

In parallel, cells were scrapped and homogenized in lysis buffer (AnaSpec, Inc., Fremont, CA, USA) in the presence of protease inhibitors (Protease Inhibitor Cocktail, Merck Life Science) for quantification of the protein content using the Pierce BCA Protein Assay Kit (Thermo-Fisher, Waltham, MA, USA). Glucose production was expressed as pg of glucose/µg of total protein/min.

### IDE activity and cAMP determination

IDE activity was assessed with the fluorometric SensoLyte® 520 IDE activity assay kit (AnaSpec, Inc., USA) as previously described by our group^14^. Briefly, cells were homogenized in 200 µL ice-cold assay buffer (AnaSpec, Inc., Fremont, CA, USA) in the presence of non-metalloprotease inhibitors (Protease Inhibitor Cocktail, Merck Life Science) plus 1 mmol/L PMSF (Merck Life Science). Homogenates were incubated on ice for 15 min, followed by centrifugation at 10,000 X *g* for 10 min at 4 °C to separate and discard insoluble materials. Supernatants were kept, and an aliquot was used for quantifying the protein content using the Pierce BCA Protein Assay Kit (Thermo-Fisher, Waltham, MA, USA). Afterward, enzymatic reactions were set up by adding 50 µL of tissue lysates in a 96-well plate. The enzymatic reaction was started by adding 50 µL of fluorogenic substrate solution into each well. The plate was gently shaken for 30 s and sample fluorescence (5-FAM) was monitored on GENios Pro TECAN multiplate reader (TECAN, Männedorf, Switzerland) every 5 min for 100 min at 37 °C. Reactions were performed in duplicate per sample. As a positive control, purified recombinant human IDE (provided by the kit) was added to the reaction mix. Wells containing the reaction mix without cell lysates were used as blanks to establish the background fluorescence levels, which were subtracted from all other readings from the same lysates. For kinetic analyses, all fluorescence readings were expressed in relative fluorescence units (RFU). RFU data were plotted versus time for each sample. Afterwards, we calculated the initial reaction velocity in RFU/min by determining the slope of the linear portion of the data plot. IDE specific activity is expressed as RFU/µg of total protein/min.

cAMP levels were quantified in cell lysates using the Cyclic AMP Competitive Elisa kit (Invitrogen, USA) following manufacturer’s protocol. The assay is based on the competition between cAMP in the standard or sample and Alkaline Phosphatase conjugated cAMP (cAMP-AP) for a limited amount of cAMP monoclonal antibody bound to an Anti-Rabbit IgG precoated 96-well plate. Briefly, Cells were serum starved overnight and preincubated in the presence or absence of glucagon for 30 min. Then, cells were harvest, resuspended in cold-PBS and lysed by ultrasonication. Lysates were centrifuged at 1500 X* g* for 10 min at 4 °C to remove cellular debris and supernatants were collected for assessing. Samples were treated with 0.1 M HCl to stop endogenous phosphodiesterase activity. Afterwards, sample dilution series in triplicate were prepared and cAMP levels were assessed by colorimetric assay (optical density at 405 nm) using a standard curve. Data were expressed as pmol/ µg of total protein.

### Plasmid construction

The cDNA for full-length human IDE (Met^1^-IDE) was obtained from the I.M.A.G.E Consortium. The cytoplasmic isoform of IDE (Met^42^-IDE) was amplified from the cDNA clone MGC:117,026 IMAGE:40,008,464 using the primers: Forward primer (5´-TTAGGATCCGCCACCATGAATAATCCAGCCATC)-3´and Reverse (5-TTTGCGGCCGCTCAGAGTTTTGCAGCCATGAAG-3´). *Ide* sequence, was cloned into a pEF1/V5-HisA vector (Invitrogen, USA). All constructions were verified by nucleotide sequencing (Genomics Unit from the Complutense University of Madrid, Spain). The plasmid was then transfected into HepG2 cells by using calcium phosphate as described elsewhere.

### Generation of shRNA-based knockdown AML12 cells

The shRNA-IDE lentiviral vector was constructed by Sigma-Aldrich (St. Louis, MO, USA), using the plasmid MISSION® pLKO.1-puro encoding the mouse shRNA-IDE sequence 5´-GCTTGCTATTAGAGAGACAAA- 3´ (TRCN0000009487). To obtain the *Ide* knockdown stable cell line, AML12 cells seeded into 6-well plate at 2 × 10^5^ cells per well. After 24 h, the lentiviral particles were added to the cells with a multiplicity of infection (MOI) of 100, and hexadimethrine bromide (polybrene; 8 µg/mL) (Sigma-Aldrich). After 96 h, the transduction efficiency was observed through a fluorescence microscope. Then, the medium was discarded, and cells were incubated with DMEM:F12 medium containing 2 μg/mL puromycin. Cells were further cultured under selection conditions, and IDE knockdown was tested through PCR and western blot analyses. Cells transduced by lentivirus with the MISSION® pLKO.1-puro empty vector (#SHC001, Sigma-Aldrich) were used as control cells.

### Generation of CRISPR‐Cas9‐based knockout HepG2 cells

HepG2-IDE-KO cells were generated using the CRISPR/Cas9 tool and a donor targeting plasmid as previously described^37^. Plasmid Construction: The donor targeting plasmid to generate the HepG2-IDE-KO cell line was obtained by cloning a blasticidin cassette and two flanking recombination arms (left and right) into an AAV-MCS vector. Both homologous arms were amplified by PCR from genomic HepG2 DNA. The left arm (primers F1/R1, Suppl. Table 1) and the right arm (primers F3/R3 in Suppl. Table 1) were homologous to a 780 bp fragment of intron 3 and a 1028 bp fragment of intron 4, respectively. The blasticidin cassette (primers F2/R2 in Suppl. Table 1) was amplified from a pBluescript II-based plasmid previously generated in the laboratory of Dr. de la Fuente.

Generation of HepG2-IDE-KO cell lines: Genetic depletion of *Ide* in HepG2 was generated using the CRISPR/Cas9 tool and a donor targeting plasmid. Guide RNAs (gRNAs) were designed using the CRISPOR software (primers F4/R4 and F5/R5, Suppl. Table 1). Two gRNA target sequences flanking the IDE exon 4 were selected to remove the complete exon. gRNAs were generated by in vitro transcription using the GeneArt Precision Synthesis Kit (Invitrogen, USA).

Briefly, 10^6^ HepG2 cells were mixed with 5 μg of Cas9 (TrueCut Cas9 Protein v2, ThermoFisher), the two gRNAs (1 μg each), and 1 μg of the donor plasmid and resuspended in 100 μL of Buffer R. Cells were nucleofected (Neon Electroporation System, ThermoFisher, USA) at a 1200 V/50 ms pulse to deliver CRISPR components into the cells. After two days, cells were grown at limiting dilution in the presence of blasticidin selection (3 μg/mL) to establish individual clones. Isolated clones were analyzed by PCR with primers external to the homologous arms and primers located in the blasticidin cassette (PCRs with F6/R6 and F7/R7 for the left and right arm respectively, Suppl. Table 1), to verify that homologous recombination had occurred and that exon 4 had been deleted. Additionally, the deletion of exon 4 was checked for heterozygosity or homozygosity by PCR (primers F8/R8, Suppl. Table 1). Genotyping results were confirmed by Sanger sequencing. The absence of IDE protein in selected clones was verified by Western blotting.

### Quantitative real-time PCR

qPCR analyses were performed as previously described^14^. Briefly, total RNA from a 80–90% confluent AML12 cell culture was isolated using TRIzol Reagent (Sigma-Aldrich, USA) following the manufacturer’s protocol. Quantification of total RNA was performed measuring ultraviolet absorbance in a NanoDrop® ND-1000 full-spectrum spectrophotometer. The removal of any potential genomic DNA contamination was achieved by treating the samples with the RapidOut DNA Removal Kit (Thermo Scientific™, Waltham, MA, USA). After DNase treatment 500–1000 ng of RNA was used to synthesize cDNA with the Transcriptor First Strand cDNA Synthesis Kit (Roche, USA). mRNA levels were determined by real time qPCR with TaqMan® probe-based assays using the 2^−ΔΔCt^ relative quantification method. TaqMan® Gene Expression assay references (from Applied Biosystems, USA) were as follows: Mm01247058_m1 for phosphoenolpyruvate carboxykinase (*Pck1*), Mm00839363_m1 for glucose-6 phosphatase (*G6pc*), Mm00433546_m1 for glucagon receptor (*Gcgr*) and Mm00501607_m1 for cAMP Response Element-Binding Protein (*Creb1*). Expression data were normalized to the level of the housekeeping gene of the ribosomal protein L18 (RPL18; Forward: 5′-AAGACTGCCGT GGTTGTGG-3′; Reverse: 5′-AGCCTTGAGGATGCGACTC-3′; Probe: 5′-FAM-TTCCCAAGCTGAAGGTGTGTGCA-BHQ1–3′).

### Western blot analysis

Western blot analyses on liver tissues (~ 20 mg) or cultured hepatocytes (primary hepatocytes or AML12 cells) were performed as previously described^38^. Briefly, Protein lysates from liver tissues or cells were prepared by homogenizing the tissues or cells in 200 µL ice-cold assay buffer (AnaSpec, Inc., Fremont, CA, USA) in the presence of non-metalloprotease inhibitors (Protease Inhibitor Cocktail, Merck Life Science) plus 1 mmol/L PMSF (Merck Life Science). Homogenates were incubated on ice for 20 min followed by sonication for 5 min on ice. Then lysates were centrifuged at 18,500 × *g* for 10 min at 4 °C to separate and discard insoluble materials. Supernatants were kept and quantified for protein content using the Pierce BCA proteins assay kit (Thermo-Fisher, USA). Afterwards, 40 µg of protein from the lysates were boiled 5 min in Laemmli Sample Buffer (LSB) (62.5 mM Tris–HCl, pH 6.8, 5% glycerol, 1% SDS, 2.5% β-mercaptoethanol and 0.02% w/v bromophenol blue). Protein samples were separated by their molecular weight using 10% SDS-PAGE polyacrylamide gel and electro-transferred onto polyvinylidene difluoride filters (PVDF; Millipore, USA) for immunoblotting by conventional means. Electro-transferred PVDF membranes were blocked for 1 h at room temperature using blocking buffer (1X PBS, 0.1% Tween-20 with 5% w/v non-fat dry milk). After being probed with specific antibodies, the membranes were stripped using stripping buffer (2% SDS, 62.5 mM Tris–HCl, pH 6.8 and 100 mM β-mercaptoethanol) for 30 min at 50 °C and then washed and reprobed with specific antibodies. A list of antibodies, commercial source, and working dilution is shown in Suppl. Table 2. Signals were detected by chemiluminescence (Clarity Western ECL Substrate, Bio-Rad™, Madrid, Spain) and exposure to X-ray film to produce bands within the linear range. Band intensity was quantified with the ImageJ software (NIH, Bethesda, MA, USA). Briefly, developed films were scanned at 600 pixels per inches with HP Scanjet G4010 (Hewlett-Packard, Madrid, Spain) using the HP Photosmart Premier 6.5 software (Hewlett-Packard, Madrid, Spain). The obtained images (negative) were converted to a 32-bit format and were inverted in order to generate an image with detectable bands. Each band was individually selected with rectangular ROI selection, followed by the quantification of the peak area of obtained histograms.

### Extraction and analysis of lipids

Diacylglycerol (DAG) extraction and analysis were performed as previously described^39–41^. Briefly, DAG was separated by thin-layer chromatography using n-hexane/diethyl ether/acetic acid (70:30:1, v/v/v) as the mobile phase. The bands corresponding to DAG were scraped off from the plate, and were subjected to transmethylation with 0.5 M KOH in methanol for 60 min at 37 °C. The resulting fatty acid methyl esters were quantified by GC–MS using an Agilent 7890A gas chromatograph coupled to an Agilent 5975C mass-selective detector operated in electron impact mode (EI, 70 eV), equipped with an Agilent 7693 autosampler and an Agilent DB23 column (60 m length × 0.25 mm internal diameter × 0.15 µm film thickness). Data analysis was carried out with the Agilent G1701EA MSD Productivity Chemstation software (Agilent Technologies, Santa Clara, CA, USA). DAG mass levels were calculated by adding the masses of its constituent fatty acids (in pmol), and dividing by 2.

### Transcriptomic microarray

Transcriptomic analysis was performed on control and HepG2-IDE-KO cells. For each experimental condition, four independent RNA replicates were processed and analyzed. RNA (1 µg) was subjected to cDNA microarray analysis on the Clarion™ S Array (Thermofisher). Data were acquired on the GeneChip™ 3000 instrument (Affymetrix) and highthroughput automated processing was performed using the GeneTitan™ Microarray System (Thermofisher). Microarray raw data were preprocessed using the Robust Multi-array Average (RMA) algorithm to normalize the data, correct for background noise and summarise across arrays using transcript clusters containing safely annotated genes. Quality control was performed by assessing the distribution of probe intensities and the presence of outliers using box plots and MA plots. All samples had good quality and none were removed from the analysis. Following preprocessing, differential gene expression analysis between Control (n = 4) and IDE-KO (n = 4) samples was conducted by fitting a linear model to the expression data for each gene and computing empirical Bayes moderated t-statistics to identify significantly differentially expressed genes (DE genes). Multiple testing correction was applied using the Benjamini–Hochberg false discovery rate (FDR) method to control for type I errors. Genes with an adjusted *p*-value < 0.05 and a log2(Fold Change) >|1| were considered significantly DE. Functional enrichment analysis was performed on the identified gene sets to explore biological pathways and gene ontology (GO) terms enriched among the DE genes. A background set of genes similar in expression to the DE genes were selected for statistical testing. Statistical significance was assessed by means of Fisher test with modifications, in order to eliminate local dependencies between GO terms and point to relevant areas in the GO graph that remain undetected.

All analyses were performed by Biostatista (ES, Palencia, https://biostatista.com) within the R statistical environment. Microarray data was managed, annotated and RMA-calibrated using the packages oligo, ArrayExpress and clariomshumanhttranscriptcluster.db. Quality control was performed using the arrayQualityMetrics package. Modelling of gene expression was performed using the limma package. GO analysis and selection of background genes were performed using the packages topGO and genefilter.

### Statistical analysis

Statistical analysis of data was performed using Prism v.6.0 (GraphPad Software). Distributions were checked with the Kolmogorov–Smirnov test. Data are presented as means ± SEM. Comparisons between two groups were done using the unpaired Students’ t-test. Comparisons between more than two groups were done using the two-way ANOVA and as a pot hoc test the two-stage setup method of Benjamini, Krieger and Yekutieli. Differences were considered significant at *p* < 0.05.

## Supplementary Information

Supplementary Information.

## Data Availability

The datasets generated and/or analyzed during the current study are available in the GEOarchive repository, Accession Number: GSE297588.

## Abbreviations

IDE: Insulin-degrading enzyme
L-IDE-KO: Mice with liver-specific deletion of *Ide*
GCGR: Glucagon receptor
GPCR: G protein coupled-receptor
PKA: Protein kinase A
CREB: CAMP-response element binding protein
p-CREB: Phosphorylated CREB
G6pc: Glucose-6-phosphatase
Pck1: Phosphoenolpyruvate carboxykinase
PLC: Phospholipase C
IP3: Inositol-1,4,5-triphosphate
DAG: Diacylglycerol
PIP2: Phosphatidylinositol-4,5-bisphosphate
HKDC1: Hexokinase domain containing 1
G6PC1: Glucose-6-phosphatase catalytic subunit 1
GNG4: G protein subunit gamma 4
PDE4: Phosphodiesterase 4D
