# Supplementary material for: Decreased expression of insulin-degrading enzyme increases gluconeogenesis and glucose production in cultured hepatocytes administered with glucagon
Source: Sci Rep. 2025 May 31;15:19168. doi: 10.1038/s41598-025-03790-2 (PMC12126513; doi:10.1038/s41598-025-03790-2)
Supplement: Supplementary file 1 — Supplementary Information. [file 41598_2025_3790_MOESM1_ESM.pdf]

Supplementary Figure 1: Diacylglycerol and fatty acids abundance in hepatocytes. AML12 cells (control and shRNA-IDE) were exposed to serum starvation for 18h followed incubation in the absence or the presence of glucagon (50ng/mL) for 1h. Afterwards, cells were collected and levels of diacylglycerol (DAG) and fatty acid (FA) methyl esters in DAG determined. **(A)** Total DAG levels, **(B)** 16:0 levels in DAG, **(C)** 16:1 levels in DAG, **(D)** 18:0 levels in DAG, and **(D)** 18:1 levels in DAG. Results are shown as the mean ± SEM of three independent experiments. No significant differences were found.

Suppl. Figure 1

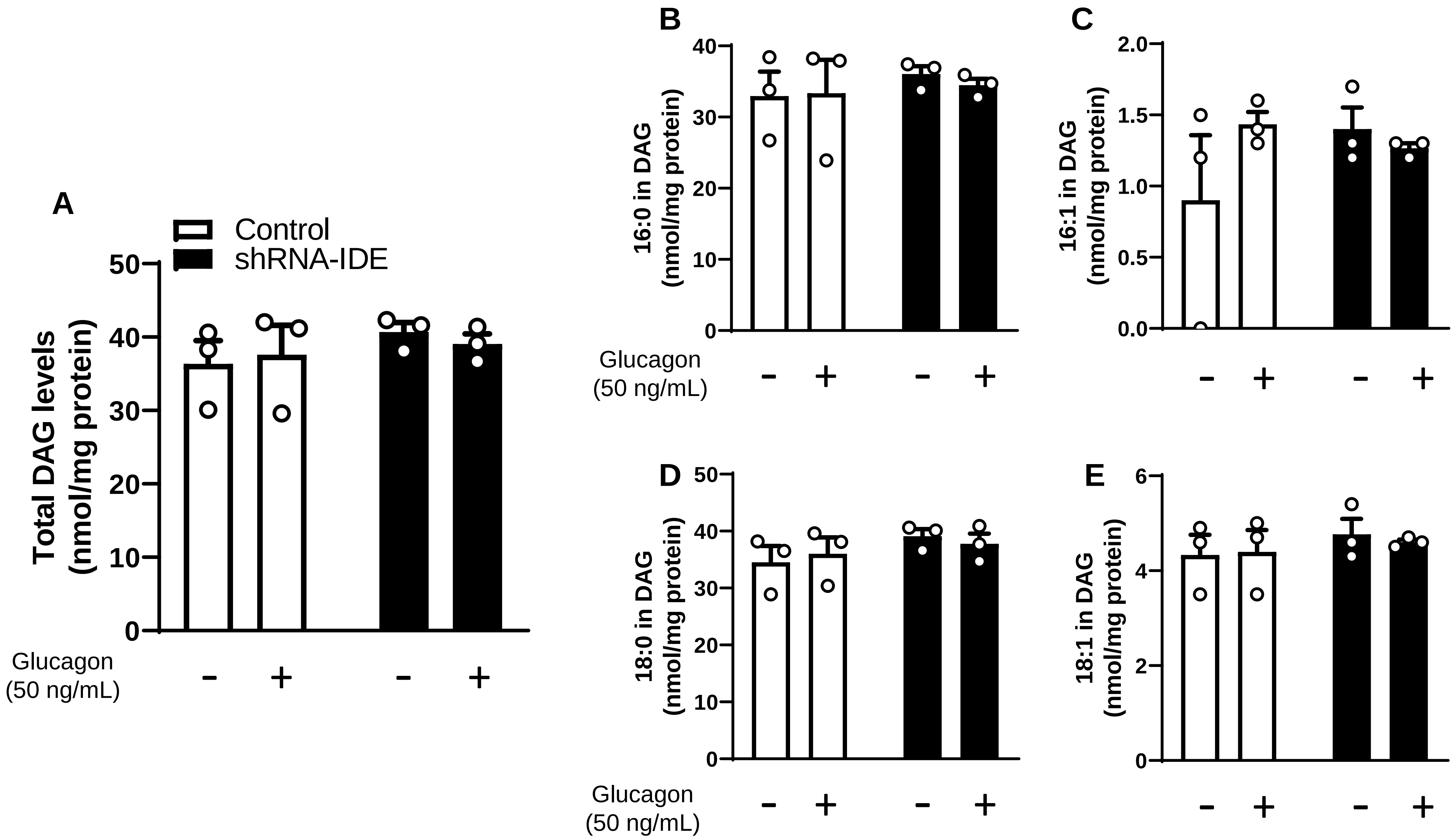

Supplementary table 1. Primers used for cloning and gRNA synthesis

Ref	Description	Sequence
F1	Left Arm NotI	5´-ATAGCGGCCGCATTATCAAGGCAAGGTTATGGG-3´
R1	Left Arm SalI	5´-AATGTCGACGATACCTACAGCTAATAAATTAAC-3´
F2	Blasticidin SalI	5´-GTCGACAATCCCGGGTCTCCGCG-3´
R2	Blasticidin EcoRI	5´-GGCGCGCCCCCAGATCGGAATT-3´
F3	Right Arm EcoRI	5´-TAAGAATTCGGCATATACAGTTCATGGCATA-3´
R3	Right Arm RsrII	5´-ATAGCGGCCGCTCACATTCCATTCTTGACATACC-3´
F4	gRNA#1 SEN	5´-TAATACGACTCACTATAGGCTGTAGGTATCTTAAAGTT-3´
R4	gRNA#1 ATS	5´-TTCTAGCTCTAAAACAACTTTAAGATACCTACAGC-3´
F5	gRNA #2 SEN	5´-TAATACGACTCACTATAGCCATGAACTGTATATGCCAT-3´
R5	gRNA #2 ATS	5´-TTCTAGCTCTAAAACATGGCATATACAGTTCATGG-3´
F6	INTRON3 EXT TEST	5´-CTGTGATGCCAAGGTGTAATTC-3´
R6	Blasticidin 5P TEST	5´-CGTTACTATGGGAACATACGTC-3´
F7	Blasticidin 3P TEST	5´-TGCCTATGCCTTATTCATCCCT-3´
R7	INTRON4 EXT TEST	5´-CCCACCTTGGTCTTCTAAAGTG-3´
F8	INTRON3 INT TEST	5´-ACTGCTATTGTCCATAATCTGCC-3´
R8	EXON4 INT TEST	5´-CCAGGCATCATTCATCACATTC-3´

Supplementary table 2. List of antibodies.

Antibody	Supplier	Catalog #	Dilution	Incubation time & ºC	Specie	MW (kDa)
**IDE**	Millipore	AB9210	1:15000	O/N 4ºC	Rabbit	110
**GCGR**	Abcam	Ab75240	1:10000	1h RT	Rabbit	54
**CREB**	Cell Signaling	9197S	1:2000	1h RT	Rabbit	43
**p-CREB**	Cell Signaling	9198S	1:2000	1h RT	Rabbit	43
**p-PKA substrates**	Cell Signaling	9624S	1:2000	O/N 4ºC	Rabbit	-
**GAPDH**	Millipore	MAB374	1:40000	1h RT	Mouse	36
**Anti-Rabbit IgG-HRP**	Jackson Immuno Research	711-035-152	1:20000	30 min RT	-	-
**Anti-Mouse IgG-HRP**	GE Healthcare	NA9310V	1:5000	30 min RT	-	-
